# Supplementary material for: Candida haemulonii complex, an emerging threat from tropical regions?
Source: PLoS Negl Trop Dis. 2023 Jul 31;17(7):e0011453. doi: 10.1371/journal.pntd.0011453 (PMC10437918; doi:10.1371/journal.pntd.0011453)
Supplement: S1 Fig — (PDF) [file pntd.0011453.s001.pdf]

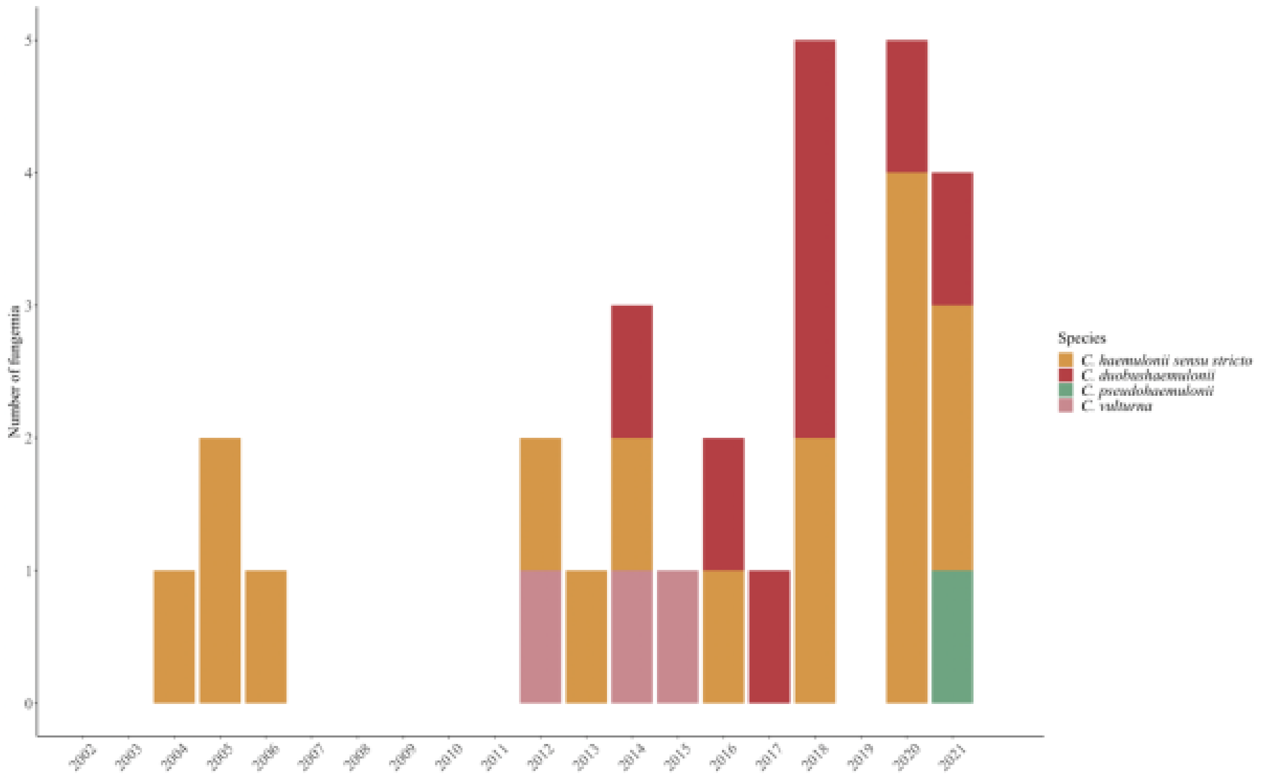

Figure S1. Bar plot representing the temporal distribution of *C. haemulonii* complex, *C pseudohaemulonii and C. vulturna* fungemia in France between 2002 and 2021 (YEASTS and RESSIF databases).
