# Supplementary material for: Candida haemulonii complex, an emerging threat from tropical regions?
Source: PLoS Negl Trop Dis. 2023 Jul 31;17(7):e0011453. doi: 10.1371/journal.pntd.0011453 (PMC10437918; doi:10.1371/journal.pntd.0011453)
Supplement: S2 Fig — (PDF) [file pntd.0011453.s002.pdf]

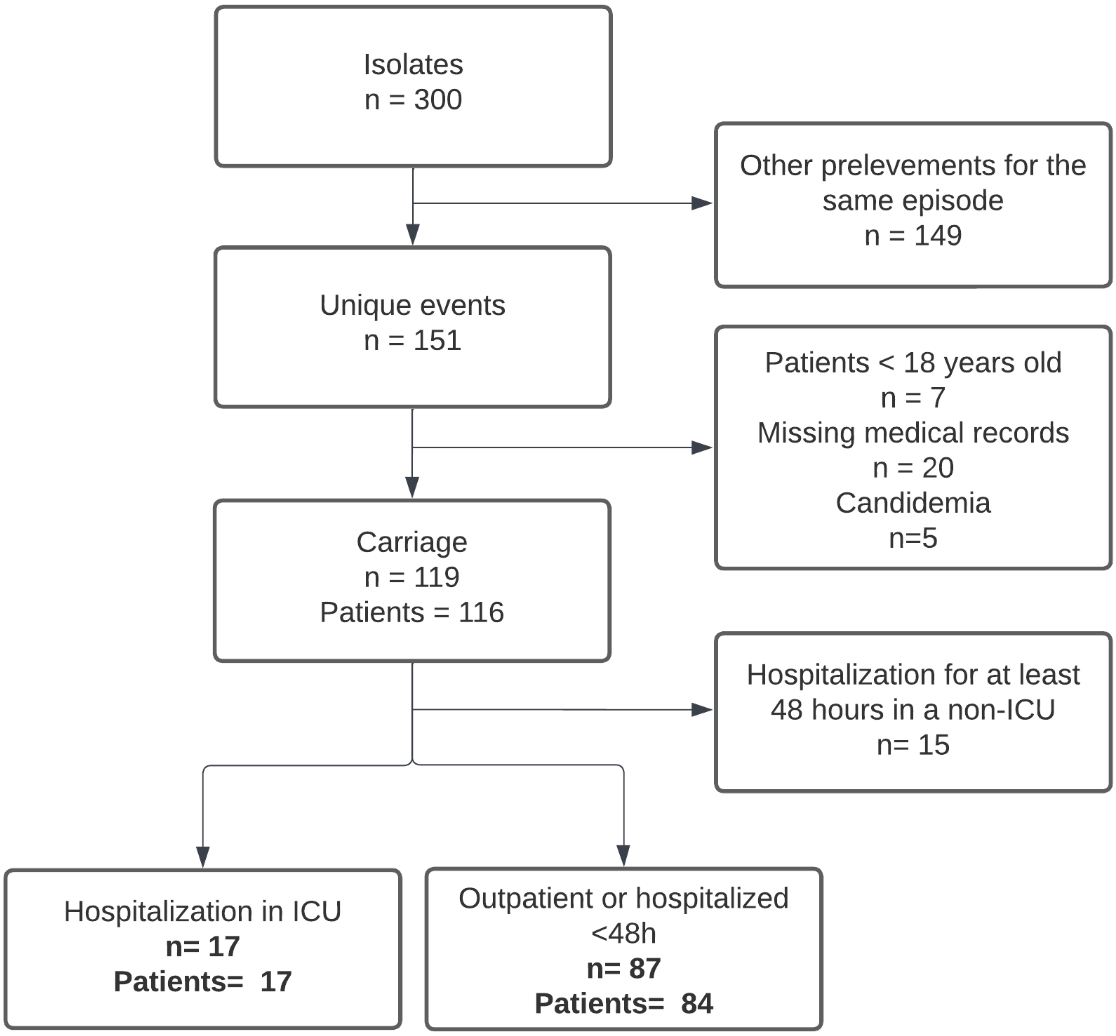

Figure S2. Flow chart of the samples with positive culture for *C. haemulonii* complex yeasts considered nonpathogenic, isolated in Martinique University Hospital (French West Indies) between 2014 and 2020.
