# Supplementary material for: Candida haemulonii complex, an emerging threat from tropical regions?
Source: PLoS Negl Trop Dis. 2023 Jul 31;17(7):e0011453. doi: 10.1371/journal.pntd.0011453 (PMC10437918; doi:10.1371/journal.pntd.0011453)
Supplement: S3 Fig — (PDF) [file pntd.0011453.s003.pdf]

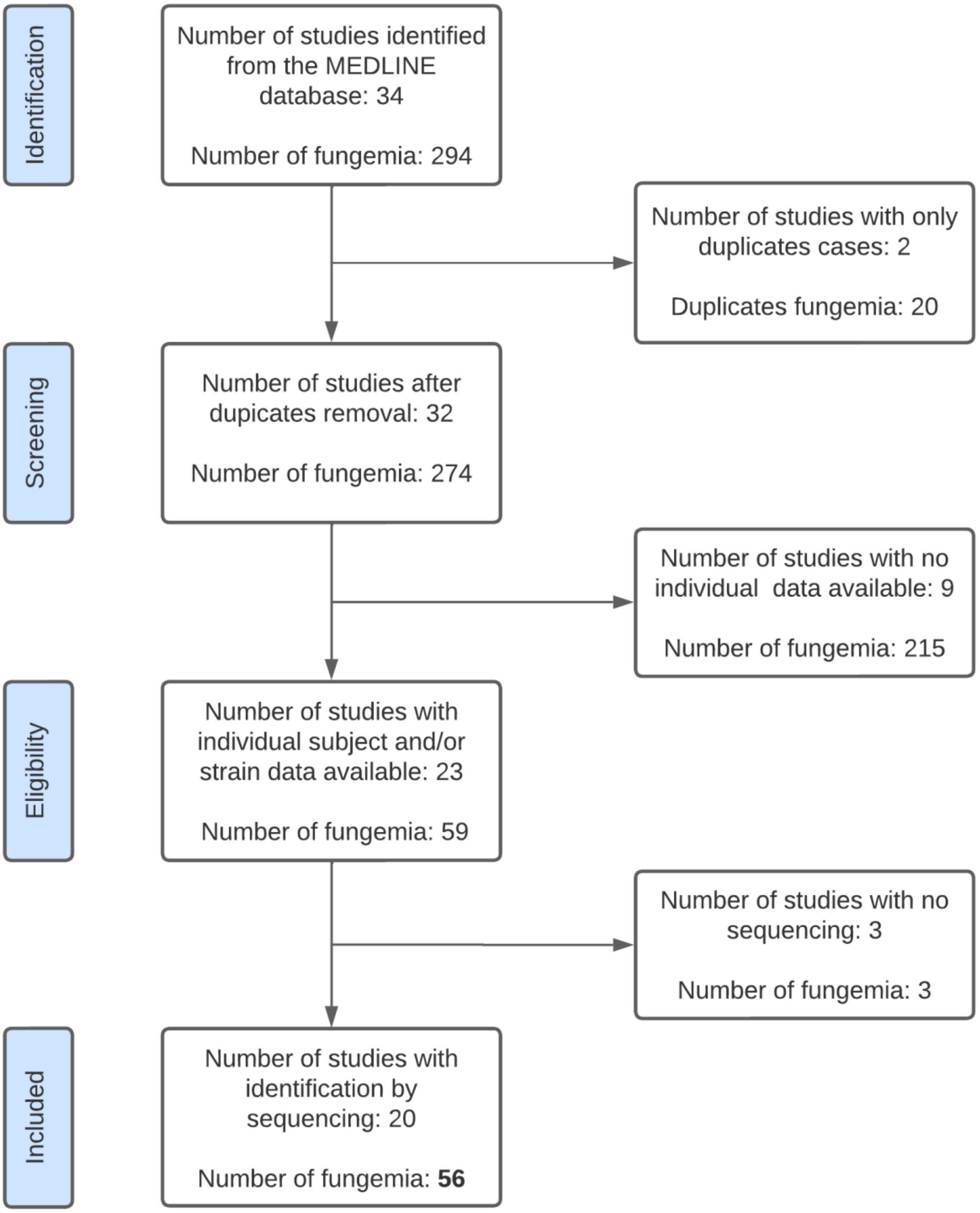

Figure S3. Flow chart of the reviewed cases of *C. haemulonii* complex, *C. pseudohaemulonii* and *C. vulturna* fungemia from MedLine (1962-2021).
References are also in the supplementary materials.
