# Supplementary material for: Candida haemulonii complex, an emerging threat from tropical regions?
Source: PLoS Negl Trop Dis. 2023 Jul 31;17(7):e0011453. doi: 10.1371/journal.pntd.0011453 (PMC10437918; doi:10.1371/journal.pntd.0011453)
Supplement: S4 Fig — (PDF) [file pntd.0011453.s004.pdf]

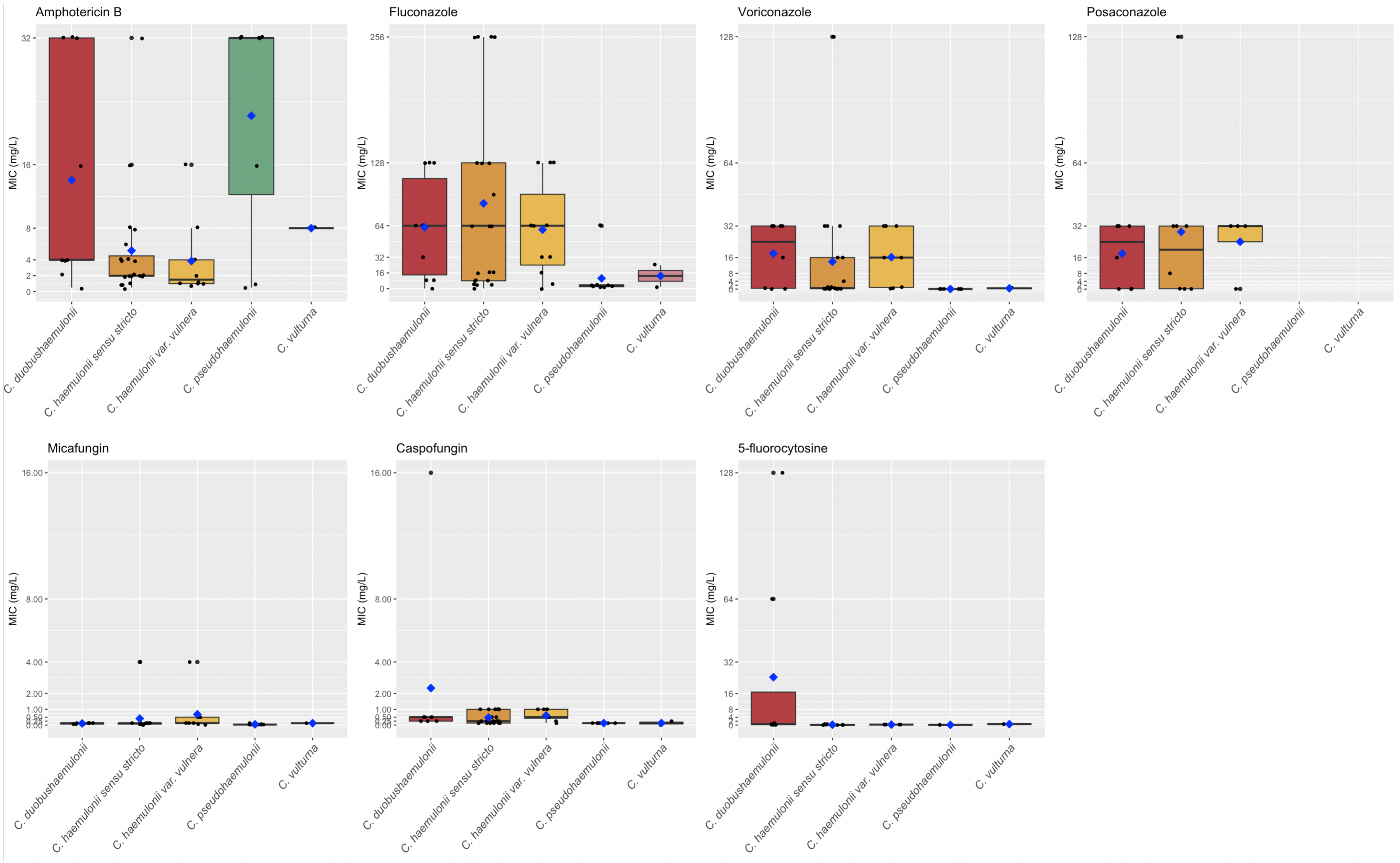

Figure S4. Susceptibility profiles of strains from the literature review (MEDLINE 1962-2022, by microdilution (n=36) or commercial methods (n=16), or not specified (n=1).

C. duobushaemulonii=10, *C. haemulonii sensu stricto*=22, *C. haemulonii var. vulnera*=11, *C. pseudohaemulonii*=8, *C. vulturna*=2

Bold black bar: median MIC value, Blue diamond: mean MIC value
