# Supplementary material for: Candida haemulonii complex, an emerging threat from tropical regions?
Source: PLoS Negl Trop Dis. 2023 Jul 31;17(7):e0011453. doi: 10.1371/journal.pntd.0011453 (PMC10437918; doi:10.1371/journal.pntd.0011453)
Supplement: S1 Table — (PDF) [file pntd.0011453.s005.pdf]

Table S1. Confirmation if identification by MALDI-TOF MS for strains initially identified complex *C. haemulonii* by API®ID32C (BioMerieux, Marcy l'Étoile, France) and stored at the University Hospital of Martinique.

Date of sampling	First correspondence	Second correspondence*	Type of sample
16/09/2014	*C. haemulonii sensu stricto*	*C. haemulonii var. vulnera*	Respiratory
14/10/2014	*C. duobushaemulonii*	-	Nail
26/12/2014	*C. duobushaemulonii*	-	Nail
24/02/2015	*C. duobushaemulonii*	-	Nail
25/02/2015	*C. duobushaemulonii*	-	Skin
24/07/2015	*C. haemulonii var. vulnera*	*C. haemulonii sensu stricto*	Contact lens
11/06/2015	*C. haemulonii sensu stricto*	*C. haemulonii var. vulnera*	Pus
24/06/2015	*C. haemulonii sensu stricto*	*C. haemulonii var. vulnera*	Nail
03/07/2015	*C. haemulonii sensu stricto*	*C. haemulonii var. vulnera*	Contact lens

*Specified if MS score >1.70
