# Supplementary material for: Candida haemulonii complex, an emerging threat from tropical regions?
Source: PLoS Negl Trop Dis. 2023 Jul 31;17(7):e0011453. doi: 10.1371/journal.pntd.0011453 (PMC10437918; doi:10.1371/journal.pntd.0011453)
Supplement: S2 Table — (PDF) [file pntd.0011453.s006.pdf]

Table S2. Characteristics of patients and strains regarding fungemia found in the literature review with individual data of *C. haemulonii* complex or related yeast fungemia identified by molecular sequencing (MedLine, 1962-2022).

Characteristic	Number
Total	56
Country (%)	
Australia	1 (2)
Brazil	33 (59)
China	2 (4)
Kuwait	4 (7)
Malaysia	3 (5)
Mexico	1 (2)
South Korea	9 (16)
Taiwan	2 (4)
Thailand	1 (2)
Median age [IQR]	10 [0.3-60.0]
Median age of the population ≥18-year-old [IQR]	66 [52-82]
Male (%)	17/32 (53)
Solid cancer (%)	7/32 (22)
Hematological malignancy (%)	5/32 (16)
Kidney failure (%)	2/30 (7)
Diabetes mellitus (%)	1/31 (3)
Corticosteroids* (%)	2/30 (7)
Other immunosuppressive treatment (%)	6/30 (20)
Surgery during the previous month (%)	5/23 (22)
Exposure to antifungal agent in the previous month (%)	9/31 (29)
Intensive Care Unit (%)	19/24 (79)
Suspected source of fungemia (%)	
Skin	30/30 (100)
Species (%)	
C. haemulonii sensu stricto	24 (43)
C. duobushaemulonii	10 (18)
C. haemulonii var. vulnera	11 (20)
C. pseudohaemulonii	9 (16)
C. vulturna	2 (4)
Antifungal treatment (%)	
Amphotericin B	10/31 (32)
Fluconazole	10/31 (32)
Amphotericin B + Fluconazole	4/31 (13)
Echinocandin	4/31 (13)
No treatment	3/31 (10)
All-cause mortality (%)	12/30 (40)

IQR: Interquartile Range
* ≥0.3 mg/kg/d for ≥ 1 month
