# Supplementary material for: Candida haemulonii complex, an emerging threat from tropical regions?
Source: PLoS Negl Trop Dis. 2023 Jul 31;17(7):e0011453. doi: 10.1371/journal.pntd.0011453 (PMC10437918; doi:10.1371/journal.pntd.0011453)
Supplement: S1 Acknowledgments — Members of the French Mycoses Study group who contributed to the data are in alphabetical order of the cities. (PDF) [file pntd.0011453.s007.pdf]

Acknowledgments

Members of the French Mycoses Study group who contributed to the data are in alphabetical order of the cities, all the French microbiologists and mycologists who sent isolates for characterization of unusual antifungal susceptibility profiles or to contribute to the ongoing surveillance program on the epidemiology of invasive fungal infections in France (YEASTS and RESSIF programs): N. Brieu (CH Aix); T. Chouaki (CHU Amiens); J. P. Bouchara, M. Pihet (CHU Angers); S. Bland (CH Annecy); V. Blanc (CH Antibes); S. Branger (CH Avignon); A. P. Bellanger, L. Millon (CHU Besançon); C. Plassart (CH Beauvais); I. Poilane (hôpital Jean Verdier, Bondy); I. Accoceberry, L. Delhaes, F. Gabriel (CH Bordeaux); A. L. Roux, V. Sivadon-Tardy (hôpital Ambroise Paré, Boulogne Billancourt); F. Laurent (CH, Bourg en Bresse); S. Legal, E. Moalic, G. Nevez, D. Quinio (CHU Brest); M. Cariou (CH Bretagne Sud); J. Bonhomme, C. Duhamel (CHU, Caen); B. Podac (CH, Chalon sur Saône); S. Lechatch (CH, Charleville-Mézières); C. Soler (hôpital d'Instruction des armées, Clamart); M. Cambon, C. Nourrisson, P. Poirier, D. Pons (CHU, Clermont Ferrand); O. Augereau, I. Grawey (CH, Colmar); N. Fauchet (CHIC, Créteil); A. Bonnin (CHU, Dijon); P. Cahen, P. Honderlick (CMC, Foch); N. Desbois, C. Miossec (CHU, Fort de France); J. L. Hermann (hôpital Raymond Poincaré, Garches) ; M. Cornet, R. Grillot, B. Lebeau, D. Maubon, (CHU, Grenoble); M. Nicolas (CHU, Guadeloupe); D. Blanchet, J. F. Carod, M. Demar, (CHU, Guyane); A. Angoulvant (hôpital Bicêtre, le Kremlin Bicêtre); C. Ciupek (CH, Le Mans); A. Gigandon (hôpital Marie Lannelongue, Le Plessis Robinson); B. Bouteille, MF Durieux (CH Limoges); D. Dupont, F. Persat (CHU, Lyon); C. Cassagne, S.Ranque (CHU, Marseille); T. Benoit-Cattin, L. Collet (CH Mayotte); A. Fiacre (CH Meaux); N. Bourgeois, L. Lachaud, Y. Sterkers (CHU, Montpellier); M. Machouart (CHU, Nancy); F. Morio (CHU, Nantes) ; O.

Moquet (CH, Nevers) ; S. Lefrançois (hôpital Américain, Neuilly) ; M. Sasso (CHU, Nimes) ; F. Reibel (GH, Nord-Essone) ; M. Gari-Toussaint, L. Hasseine (CHU Nice) ; L. Bret, D. Poisson (CHR Orléans) ; S. Brun (hôpital Avicenne, Paris) ; C. Bonnal, C. Chochillon (hôpital Bichat, Paris) ; A. Paugam (hôpital Cochin, Paris) ; N. Ait-Ammar, F. Botterel, R. Chouk (CHU Henri Mondor, Paris), M. E. Bougnoux, F. Lanternier, E. Sitterle (hôpital Necker, Paris), A. Datry, A. Fekkar (hôpital Pitié Salpêtrière, Paris); J.-L. Poirot (hôpital St Antoine, Paris) ; S. Bretagne, A. Alanio, M. Gits-Muselli, S. Hamane, C. Lacroix (hôpital Saint Louis, Paris) ; S. Bonacorsi, P. Mariani (hôpital Robert Debré, Paris) ; D. Moissenet (hôpital Trousseau, Paris) ; C. Kauffmann-Lacroix, A. Minoza, E. Perraud, (CHU Poitiers) ; G. Colonna (CH, Porto Vecchio) ; A. Huguenin, D. Toubas (CHU Reims), S. Chevrier, J. P. Gangneux, F. Robert-Gangneux (CHU Rennes); O. Belmonte, G. Hoarau, M. C. Jaffar Bandjee, J. Jaubert, S. Picot, N. Traversier (CHU Réunion); L. Favennec, G. Gargala (CHU, Rouen) ; N. Godineau, C. Tournus (CH, St Denis) ; C. Mahinc, H. Raberin (CHU, St Etienne) ; V. Letscher Bru (CHU, Strasbourg) ; S. Cassaing (CHU, Toulouse) ; P. Patoz (CH Tourcoing); E. Bailly, J. Chandenier, G. Desoubeaux (CHU Tours) ; F. Moreau (CH Troyes) ; P. Munier (CH Valence) ; E. Mazars (CH Valenciennes) ; O. Eloy (CH Versailles) ; E. Chachaty (Institut Gustave Roussy, Villejuif); A. Bertho, C. Blanc, A. Boullié, E. Fruquière, D. Garcia- Hermoso, C. Gautier, V. Geolier, D. Hoinard , D. Raoux-Barbot, and F. Dromer (NRCMA (Institut Pasteur, Paris).
