# Supplementary material for: Candida haemulonii complex, an emerging threat from tropical regions?
Source: PLoS Negl Trop Dis. 2023 Jul 31;17(7):e0011453. doi: 10.1371/journal.pntd.0011453 (PMC10437918; doi:10.1371/journal.pntd.0011453)
Supplement: Attachment — Submitted filename: Answers to reviewers.docx [file pntd.0011453.s009.docx]

## Answer to reviewers

**Methods**

-Are the objectives of the study clearly articulated with a clear testable hypothesis stated?

-Is the study design appropriate to address the stated objectives?

-Is the population clearly described and appropriate for the hypothesis being tested?

-Is the sample size sufficient to ensure adequate power to address the hypothesis being tested?

-Were correct statistical analysis used to support conclusions?

-Are there concerns about ethical or regulatory requirements being met?

Reviewer #1: Objectives of the study are clearly stated. Study design is appropriate for the stated objectives. Population described clearly and appropriate for objectives of study. Sample size is small but this is a rare disease so I think the author have done well in combining data from multiple years as well as performing a literature review. This has added weight to the findings and helped overcome the challenge of small numbers. No ethical concern.

Authors: Thank you very much for your comment, indeed we have tried to explore all available information to describe as best as possible these yet rare invasive infections. We are delighted to know how these different approaches have allowed you to better understand the issue, despite our limited C. haemulonii complex fungemia series.

Reviewer #2: The objective of the work is clear, with this research it was expected to know a little more about the C. haemulonii complex and the species that are included in it. It is a work of many years and that implied a complete experimental design, which is reflected here. All methodologies were adequate to meet the objective of the work. It should be noted that to contrast the results, epidemiological data from the region were used, which were obtained in different hospitals and foundations, in addition, these data were compared with what has already been reported in the literature. There were many samples of patients analyzed, therefore, the population n is sufficient for the statistical analyzes to be more complete. With respect to bioethical permits, initially they mention the institutions by which the work was approved, however, nothing is mentioned about the informed consent of the patients, or if this was not required.

Authors: Regarding protection of data, the database is located at the Institut Pasteur and benefits from a notification at the Commission Nationale Informatique et Libertés. For such studies, the informed consent of patients is not required and only the lack of opposition which was the case.

Reviewer #3: -Are the objectives of the study clearly articulated with a clear testable hypothesis stated? Yes.

-Is the study design appropriate to address the stated objectives? Yes.

-Is the population clearly described and appropriate for the hypothesis being tested? Yes.

-Is the sample size sufficient to ensure adequate power to address the hypothesis being tested? Yes, though samples sized were relative small.

-Were correct statistical analysis used to support conclusions? Yes.

-Are there concerns about ethical or regulatory requirements being met? No.

Authors: Thank you for your answers.

--------------------

**Results**

-Does the analysis presented match the analysis plan?

-Are the results clearly and completely presented?

-Are the figures (Tables, Images) of sufficient quality for clarity?

Reviewer #1: Analysis matches the presented plan. Results are very clear and no issues with the tables.

- Please highlight the major differences in the two groups from Table 1.

- Line 198 - Is the present of that yeast in the eye only a colonizer? Could it have a role to play in the patient's keratitis/corneal abscess?

Authors:

- Thank you for your comment. As suggested, we have bolded the significant differences between the 2 groups in Table 1.
- It is actually difficult to formally affirm the non-pathogenic character of theses yeasts. Regarding the present series, we did not retain the pathogenic character for 2 reasons: First, we followed the decision of the clinicians, who in all cases considered the presence of the yeast as non-pathogenic, and second, a known pathogen was found in all cases and was the sole the only target of the antimicrobial treatment proposed to the patients. Considering these elements, it seemed even more difficult to affirm or even suggest the pathogenic nature of *C. haemulonii* complex yeasts in these cases of corneal keratitis/abscess.

Reviewer #2: The data analysis is understandable, the tables are a good complement to understand what was found. As has been previously reported in other studies, the incidence of candidiasis increases especially in immunocompetent and immunosuppressed patients, an example of this is what was found in this study. Cancer and diabetes mellitus are diseases that are associated with an increase in candidiasis caused by these species of the complex. All the findings are well explained and are supported by tables and figures. I suggest that the figures and tables found in supplementary material be revised and modified, since they are of poor quality and much information is lost.

Authors: Thank you for your feedback. As suggested, we have updated the elements in the appendix, which indeed were not as clear as those in the article.

Reviewer #3: -Does the analysis presented match the analysis plan? Yes.

-Are the results clearly and completely presented? Yes.

-Are the figures (Tables, Images) of sufficient quality for clarity? Yes.

Authors: Thank you for your answers.

--------------------

**Conclusions**

-Are the conclusions supported by the data presented?

-Are the limitations of analysis clearly described?

-Do the authors discuss how these data can be helpful to advance our understanding of the topic under study?

-Is public health relevance addressed?

Reviewer #1: The conclusions are supported by the presented data. Limitations of the study are clearly described. This is helpful from a public health standpoint and a significant contribution to the understanding of this rare group of yeast that used to be misidentified as C. auris by some identification systems.

Authors: Thank you very much for your comment, we are glad to see that the message of our article was clear and of interest to you.

Reviewer #2: With respect to the conclusions section, I consider that these should be mentioned in the final part of the text. In the discussion section some limitations of the work are mentioned and how they could be solved, however, in the literature there is a lot of information that could help to complement this section. In the attached file there are some comments on this specific section. At the end of the paper it is necessary to mention the future perspectives of this work, why it is important to study these species, especially in the hospital environment, and the implications it has on public health.

Authors: As rightly suggested, we have added a conclusion section, mentioning the future perspectives of this work, the interest in studying these species, and the importance of epidemiological surveillance

Reviewer #3: -Are the conclusions supported by the data presented? Yes.

-Are the limitations of analysis clearly described? Yes.

-Do the authors discuss how these data can be helpful to advance our understanding of the topic under study? Yes.

-Is public health relevance addressed? Yes.

Authors: Thank you for your answers.

--------------------

Editorial and Data Presentation Modifications?

Use this section for editorial suggestions as well as relatively minor modifications of existing data that would enhance clarity. If the only modifications needed are minor and/or editorial, you may wish to recommend “Minor Revision” or “Accept”.

Reviewer #1: Minor revisions as suggested in the textboxes above and below.

Authors: Thank you for your comments.

Reviewer #2: Throughout the following text I mention some points that should be taken into account for this revision. The changes are minor. Special attention should be paid to the spelling of the reference section.

Authors: Thank you for your answers. We have revised the spelling of the references as suggested

Reviewer #3: (No Response)

--------------------

Summary and General Comments

Use this section to provide overall comments, discuss strengths/weaknesses of the study, novelty, significance, general execution and scholarship. You may also include additional comments for the author, including concerns about dual publication, research ethics, or publication ethics. If requesting major revision, please articulate the new experiments that are needed.

Reviewer #1: - First page - Abstract/Results: a psace is missing "fungemiadue" and "werefrequently"

Authors: These spaces appear in our version. It is probably a problem during formatting. This will be reviewed with the editor if needed.

- Long form of ICU needs to be provided before it can be used as an acronym (line 107)

Authors: With all due respect, I think this abbreviation is explained on line 87

- I would mention that this specie could be a C. auris misidentification on identification by Vitek 2 version 8.01

Authors: We added this precision as suggested in the introduction (line 75)

- I suggest adding the following reference to line 255-256 : Microorganisms. 2020 Feb; 8(2): 181.

Published online 2020 Jan 28. doi: 10.3390/microorganisms8020181 PMCID: PMC7074697 PMID: 32012865

Authors: thank you for this remark, we added the reference here, moreover we already quote this article in the introduction.

- I suggest reviewing "effraction" on line 267 as I am familiar with it as a francophone but this term is not commonly used in the English language to mean what its intended meaning in French

Authors: Thank you for this comment. We have replaced it with “rupture”.

Reviewer #2: General comments are available in the following text. I consider it to be an interesting and novel text, as there are few works that deal with all species of the C. haemulonii complex. In addition, it is the first report of these species in France, and to achieve this it was necessary to review biological samples from 2002 to 2021, which indicates that it is a very complete report. Contributing to the knowledge of these species has important implications for science, thanks to these studies it is possible to search for new alternative treatments for the control of these species. In addition, this information provides interesting data about the biology of the species of this complex, from their lifestyle in the environment to how they infect their host. It would be interesting to emphasize the identification of these species, since it is known that distinguishing between species is still a complicated task and in some cases unsuccessful.

Authors:

- About comments Line 180: The higher occurrence of these infections in tropical regions does not seem to be related to the medical context of the patients, as shown in the model. In contrast, living in a tropical environment, notably in the Caribbean/Latin America, is associated with the occurrence of these infections in people with a high risk of candidiasis. This occurrence is therefore likely related to the greater presence of these yeasts in these environments, as suggested by the literature, as well as to skin carriage, shown in the colonization section, which probably constitutes a pre-existing stage of the infection. We have completed the discussion in order to better explain our hypothesis (line 246-250)
- About comments Line 186 (Why compare these results with the species C. parapsilosis?): Since the suspected pathophysiology of C. haemulonii fungemia is mediated by skin carriage, it seemed more appropriate to use C. parapsilosis (and not Candida albicans we previously used in prior studies) as a comparator because the latter is a commensal yeast of the skin, and these two pathogens would therefore share a similar pathophysiology. This choice is justified like this in the methods.
- About the comments Line 235 (Why is the tropics associated with the incidence of these fungemias?): Our work does not allow us to answer this question, so we can only speculate. Yeasts of the *C. haemulonii* complex are probably more present in tropical environments, which are favorable to their development, as suggested in the literature. In the absence of specific environmental studies, it is difficult to affirm this hypothesis. As for the comments line 180, we have completed the discussion in order to better explain our hypothesis
- About the Comments line 261 (What reports are there for C. auris?): Similar results have been reported in the literature concerning C. auris, which can colonize medical devices. This is discussed in the article by Sabino et al. so we have added the reference for this paragraph.
- About the comments line 265 (Is there any strategy to improve these limitations?):
  - Missing data: All cases with missing data were re-examined by the biologists of the concerned centers. The remaining missing data might have been found in the complete patient records, we did not have access to them.
  - Skin carriage: in the absence of evidence of concomitant skin carriage with fungemia in our series, we can only suspect it. Performing fungal skin mapping in patients with risk factors of fungemia (catheter, ICU, cancer...) could highlight this carriage and consequently adapt the probabilistic treatment.
- About the comments line 288 (At the end of the text it would be important to mention the prospects for the future with this work and what is the importance of studying these species): The conclusion section that we have added according to your suggestion, allows us to support these elements.
- About the comments line 457 (This figure is not mentioned in the text): With all due respect, I think the Figure S3 is mentioned line 189.

Reviewer #3: General comments

1. I review this manuscript entitled “Candida haemulonii complex, an emerging threat from tropical regions?” with interest. This manuscript described clinical and demographic characteristics of patients with fungemia due to C. haemulonii complex and related species (C. pseudohaemulonii, C. vulturna).

2. This is a neglected tropical disease warrants more attention because C. haemulonii complex (C. haemulonii sensu stricto, C. duobushaemulonii, C. haemulonii var. vulnera) and related species (C. pseudohaemulonii and C. vulturna), are phylogenetically close to Candida auris with intrinsic antifungal resistance. I fully agree what authors emphasized “Multidrug-resistant C. haemulonii complex-related species are responsible for fungemia and colonization in community and hospital settings, especially in tropical regions, warranting closer epidemiological surveillance to prevent a potential C. auris-like threat.” (Abstract, Conclusions/Significance). However, its importance is underestimated due to difficulty in accurate identification and thus, underreported.

3. Due to limited case numbers and published data, this manuscript included three part of results. A nationwide case series reported in France during 2002-2021 (28 cases, the first epidemiological data on these yeasts in France), a single center study regarding colonization and infection due to C. haemulonii complex (40 cases), and a literature review of fungemia due to C. haemulonii complex and related species reported in Medline (1962-2022) (274 cases). The final part included all reported cases of C. haemulonii complex, C. pseudohaemulonii, and C. vulturna fungemia identified by sequencing, for which individual clinical information and/or susceptibility to antifungals were available.

Specific comments

Lines 158-160, please shorten as these data were presented in the Table 1.

Authors: Thank you for your comment. We have removed the redundant numbers to make it easier to read.

Lines 161-163, “Four distinct species were identified….”, thus, suggest delete the same content in Table 1.

Authors: As suggested, we have removed the corresponding lines in Table 1.

Table 1. Among patients with available birth continent 18 of 20 (90.0%) patients with C. haemulonii complex-related species were America. Is it due to the majority of cases were reported from oversea France?

Authors: Yes, since the vast majority of patients in hospitals in the French overseas territories live in these regions, it explains this result.

--------------------

PLOS authors have the option to publish the peer review history of their article (<a href="<https://journals.plos.org/plosntds/s/editorial-and-peer-review-process#loc-peer-review-history>" target="_blank">what does this mean?</a>). If published, this will include your full peer review and any attached files.<br><br>

If you choose “no”, your identity will remain anonymous but your review may still be made public.<br><br>

<b>Do you want your identity to be public for this peer review?</b> For information about this choice, including consent withdrawal, please see our <a href="<https://www.plos.org/privacy-policy>" target="_blank">Privacy Policy</a>.

Reviewer #1: Yes: Samuel Bourassa-Blanchette

Reviewer #2: Yes: Manuela Gómez Gaviria

Reviewer #3: No

Figure Files:

While revising your submission, please upload your figure files to the Preflight Analysis and Conversion Engine (PACE) digital diagnostic tool, [https://pacev2.apexcovantage.com](https://pacev2.apexcovantage.com/). PACE helps ensure that figures meet PLOS requirements. To use PACE, you must first register as a user. Then, login and navigate to the UPLOAD tab, where you will find detailed instructions on how to use the tool. If you encounter any issues or have any questions when using PACE, please email us at figures@plos.org.

Data Requirements:

Please note that, as a condition of publication, PLOS' data policy requires that you make available all data used to draw the conclusions outlined in your manuscript. Data must be deposited in an appropriate repository, included within the body of the manuscript, or uploaded as supporting information. This includes all numerical values that were used to generate graphs, histograms etc.. For an example see here: <http://www.plosbiology.org/article/info%3Adoi%2F10.1371%2Fjournal.pbio.1001908#s5>.

Reproducibility:

To enhance the reproducibility of your results, we recommend that you deposit your laboratory protocols in [protocols.io](http://protocols.io/), where a protocol can be assigned its own identifier (DOI) such that it can be cited independently in the future. Additionally, PLOS ONE offers an option to publish peer-reviewed clinical study protocols. Read more information on sharing protocols at <https://plos.org/protocols?utm_medium=editorial-email&utm_source=authorletters&utm_campaign=protocols>

References

Please review your reference list to ensure that it is complete and correct. If you have cited papers that have been retracted, please include the rationale for doing so in the manuscript text, or remove these references and replace them with relevant current references. Any changes to the reference list should be mentioned in the rebuttal letter that accompanies your revised manuscript. If you need to cite a retracted article, indicate the article's retracted status in the References list and also include a citation and full reference for the retraction notice.
